# On‐treatment dynamics of circulating extracellular vesicles in the first‐line setting of patients with advanced non‐small cell lung cancer: the LEXOVE prospective study

**DOI:** 10.1002/1878-0261.13737

**Published:** 2025-01-09

**Authors:** Valerio Gristina, Viviana Bazan, Nadia Barraco, Simona Taverna, Mauro Manno, Samuele Raccosta, Anna Paola Carreca, Marco Bono, Tancredi Didier Bazan Russo, Francesco Pepe, Pasquale Pisapia, Lorena Incorvaia, Giuseppe Badalamenti, Giancarlo Troncone, Umberto Malapelle, Daniele Santini, Antonio Russo, Antonio Galvano

**Affiliations:** ^1^ Department of Precision Medicine in Medical, Surgical and Critical Care (Me.Pre.C.C.) University of Palermo Italy; ^2^ Department of Experimental Biomedicine and Clinical Neurosciences University of Palermo Italy; ^3^ Institute of Translational Pharmacology (IFT) National Research Council (CNR) of Italy Palermo Italy; ^4^ Institute of Biophysics (IBF) National Research Council (CNR) of Italy Palermo Italy; ^5^ Ri.MED Foundation Palermo Italy; ^6^ Department of Public Health University of Naples Federico II Italy; ^7^ Medical Oncology A, Policlinico Umberto 1 La Sapienza Università Di Roma Italy

**Keywords:** DLS, dynamics, EVs, liquid biopsy, NSCLC

## Abstract

Extracellular vesicle (EV) monitoring can complement clinical assessment of cancer response. In this study, patients with advanced non‐small cell lung cancer (NSCLC) undergoing osimertinib, alectinib, pembrolizumab or platinum‐based chemotherapy ± pembrolizumab were enrolled. EVs were characterized using Bradford assay to quantify the circulating cell‐free EV protein content (cfEV), and dynamic light scattering to assess Rayleigh ratio excess at 90°, z‐averaged hydrodynamic diameter and polydispersity index. A total of 135 plasma samples from 27 patients were collected at baseline (T0) and at the first radiological restaging (T1). A ∆cfEV < 20% was associated with improved median progression‐free survival (mPFS) in responders versus non‐responders. Specifically, cfEV responders on pembrolizumab had a significantly better mPFS (25.2 months) compared to those on chemotherapy plus pembrolizumab (6.1 months). *EGFR*‐positive cfEV responders also experienced longer mPFS compared to cfEV non‐responders (35.1 months, 95% CI: 14.9–35.5 vs. 20.8 months, 95% CI: 11.2–30.4). This study suggested that monitoring circulating EV could provide valuable insights into treatment efficacy in NSCLC, particularly for patients receiving pembrolizumab or osimertinib.

## Graphical abstract

The LEXOVE prospective study evaluated plasma cell‐free extracellular vesicle (cfEV) dynamics using Bradford assay and dynamic light scattering in metastatic non‐small cell lung cancer patients undergoing first‐line treatments, correlating a ∆cfEV < 20% with improved median progression‐free survival in responders versus non‐responders. This highlights the potential of cfEVs as valuable liquid biopsy biomarkers for assessing treatment efficacy.

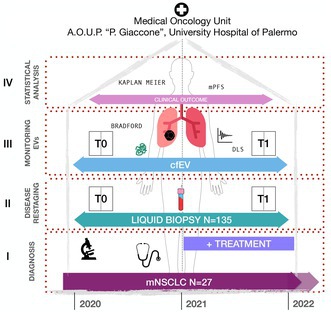

**Abbreviations**
(Mw)_2_
: squared weight‐averaged mass
AIFA: Agenzia Italiana del Farmaco
ALIX = PDCD6IP: programmed cell death 6‐interacting protein
ALK: anaplastic lymphoma kinase
BA: Bradford assay
BRAF: serine/threonine‐protein kinase B – rapidly accelerated fibrosarcoma
CD63: cluster of differentiation 63
CD81: cluster of differentiation 81
cfDNA: circulating cell‐free DNA
cfEV: cell‐free EV protein content
CI: confidence interval
CR: complete response
CT: platinum‐based chemotherapy
DLS: dynamic light scattering
DNA: deoxyribonucleic acid
Dz: z‐averaged hydrodynamic diameter
ECOG: Eastern Cooperative Oncology Group
EGFR: epidermal growth factor receptor
EVs: extracellular vesicles
FFPE: formalin‐fixed paraffin‐embedded
HGVS: Human Genome Variation Society
HPLC‐SEC: high‐performance liquid chromatography—size exclusion chromatography
IASLC: International Association for the Study of Lung Cancer
IHC: immunohistochemistry
INNOVA: Italian Network of excellence for advanced diagnosis
IQR: interquartile range
KRAS: Kirsten rat sarcoma
LEXOVE: lung extracellular vesicles study
MET: mesenchymal‐epithelial transition factor
mPFS: median progression‐free survival
NA: nucleic acids
NGS: next‐generation sequencing
NSCLC: non‐small cell lung cancer
NTRK: neurotrophic tyrosine receptor kinase
Osi: osimertinib
*P*(*D*_h_): distribution function of the hydrodynamic diameter
*P*(*q*): theoretical scattering form factor
PD: progressive disease
PDI: polydispersity index
Pembro: pembrolizumab
PFS: progression‐free survival
PNRR: Piano Nazionale di Ripresa e Resilienza project
PR: partial response
PS: performance status
R90: Rayleigh ratio excess at angle 90°
RECIST: response evaluation criteria in solid tumors
RET: rearranged during transfection proto‐oncogene
RNA: ribonucleic acid
ROS1: ROS proto‐oncogene 1, receptor tyrosine kinase
RT‐PCR: real‐time‐polymerase chain reaction
SD: stable disease
SLS: static light scattering
T0: timepoint 0 = baseline
T1: timepoint 1 = first radiologic disease restaging
TBS‐T: tris‐buffer saline, 0.1% tween
TNM: tumor node metastasis staging
TPS: tumor proportion score
TSG‐101: tumor susceptibility gene 101
W12: first disease restaging at 12 weeks
WB: western blot
Δ cfEV: cell‐free EV protein levels from baseline to disease restaging

## 1 Introduction

Despite the increasing availability of diagnosis and personalized therapeutic approaches, most patients with non‐small cell lung cancer (NSCLC) usually present with an advanced stage of disease, regrettably showing very low 5‐year survival rates [1]. Although several biomarkers have been described in this complex scenario [2, 3, 4], there remains an unmet clinical need for the discovery of dynamic *in vivo* biomarkers to refine the clinical management of such patients, predicting response and prognostics to eventually improve the treatment sequencing while avoiding ineffective therapies.

Over the last decade, emphasis has been placed on nano/micrometer‐sized vesicles for their role in intercellular communications contributing to tumor growth, metastasis, angiogenesis, and drug resistance [5]. Extracellular vesicles (EVs) are a heterogeneous group of double membrane‐enclosed vesicles released by all cytotypes in physiological and pathological conditions [6]. EVs are classified based on their size, origin, and release mechanism [7, 8, 9, 10], shuttling a plethora of bioactive molecules such as proteins, lipids, metabolites, and nucleic acids [11, 12, 13].

In the liquid biopsy era, albeit the circulating cell‐free DNA (cfDNA) analysis remains the gold standard for routine clinical diagnostics [14], the evaluation of EV amount and morphology might be a complementary tool to assess response and guide the clinical decision‐making process [15]. Accordingly, a growing body of evidence has proved the utility of circulating EVs as minimally invasive biomarkers in different disease settings, even if often at the preclinical level [16, 17]. Compelling evidence suggests that the anti‐tumor response is a multifaceted process regulated by intricate interactions among the tumor, the immune system, and various host factors in which EVs seem to hold a pivotal role [18, 19].

It is firmly established that patients with cancer present with a higher proportion of circulating EVs when compared to healthy subjects [20, 21]. Cancer‐associated EVs, presenting with a favorable lipid bilayer structure with high biocompatibility and inherent targeting ability, have proved to be promising diagnostic and prognostic biomarkers in cancer patients, showing great potential for endorsing hierarchical management and monitoring clinical outcomes [20, 21, 22]. Therefore, clinical cohort trials in real‐life settings are needed to evaluate if serial sampling of plasma EVs may provide real‐time assessment of therapeutic responses while enabling the on‐treatment monitoring of patients with advanced NSCLC.

From this perspective, differing methods of EV isolation and characterization have been set out with dynamic light scattering (DLS) representing a promising technique for determining the particle size distribution in a colloidal suspension such as human plasma [23]. Making use of the Brownian motion of suspended particles in a solvent, DLS would enable the analysis of nanoparticles by evaluating the hydrodynamic diameter (Dz) and the Rayleigh scattering (R90) to compare the concentration of equally sized and shaped nanoparticles [24]. Furthermore, the quantification and dynamics of EV biophysical properties and total protein content during first‐line systemic treatments have never been prospectively evaluated as a potential clinical tool in NSCLC.

In this explorative study, we aimed to describe whether the serial evaluation of plasma tumor‐derived EVs could longitudinally reflect response and resistance to available first‐line treatments, investigating the potential to predict clinical outcomes in patients with advanced NSCLC.

## 2 Materials and methods

### 2.1 Institutional Review Board statement

The study was conducted according to the guidelines of the Declaration of Helsinki and approved by Palermo 1 Institutional Ethic Review Board (Statement No. 02/2020, approved on 19 February 2020, AIFA code CE 150109). Informed consent was obtained from all subjects involved in the study.

### 2.2 Study design and patients

**Fig. 1:**
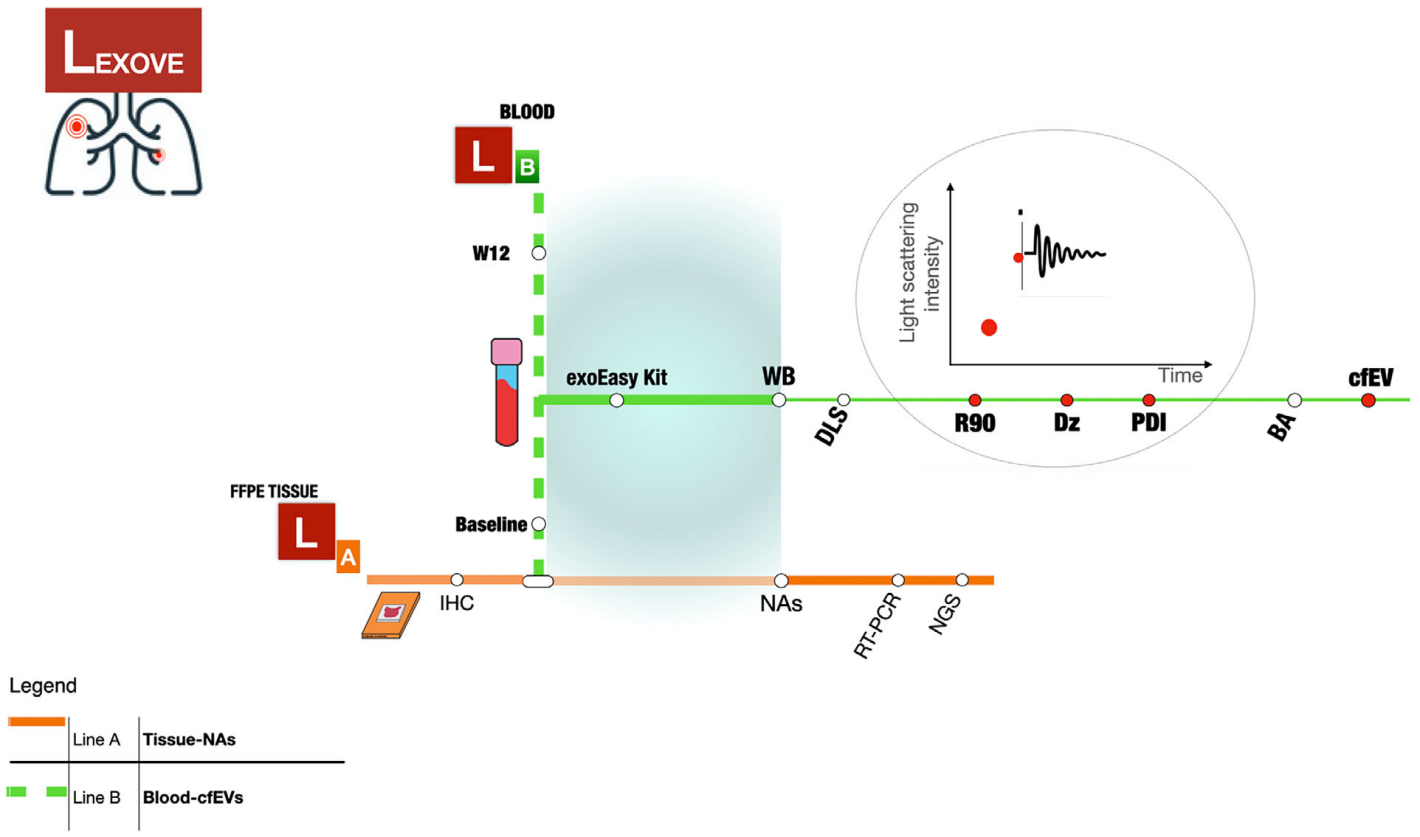
Graphical scheme design of the LEXOVE prospective study. Line A shows patients' accrual according to tissue predictive molecular pathology following clinical practice guidelines. Line B depicts complementary evaluation of plasma samples for extracellular vesicles (EVs) analyses at baseline and the first radiologic evaluation of disease within 12 ± 1 weeks (W12): circulating EV isolation was carried out using serial centrifugation steps followed by a membrane affinity‐based commercial method (exoEasy‐kit); isolated EVs were characterized using either western blot (WB) to determine EV‐based markers, Dynamic Light Scattering (DLS) to assess the amount and the size distribution of EVs (R90, Rayleigh ratio excess at angle 90°; Dz, z‐averaged hydrodynamic diameter; PDI, polydispersity index) or Bradford assay (BA) for the quantification of circulating EV protein content (cfEV, cell‐free EV). FFPE, formalin‐fixed paraffin‐embedded; IHC, immunohistochemistry; NA, nucleic acids; NGS, next‐generation sequencing; RT‐PCR, real‐time‐polymerase chain reaction.

The Lung EXtracellular VEsicles (LEXOVE) study is a prospective cohort observational trial aiming to investigate circulating EVs as potential and minimally invasive biomarkers for monitoring and prognostics of treatment‐naïve patients with advanced NSCLC undergoing standard first‐line treatments (Fig. 1). From February 2020 to May 2022, patients with advanced NSCLC treated at the Medical Oncology Unit of Paolo Giaccone University Hospital of Palermo, Italy, were consecutively enrolled. Paired blood samples were collected at baseline (T0) and the first radiologic evaluation of disease within 12 ± 1 weeks (T1 or W12) during the treatment course. The collected plasma samples were used to isolate EVs that were characterized by DLS and Bradford assay (BA). All the patients underwent a computerized tomography scan every 3 months and radiologic responses were classified according to the Response Evaluation Criteria in Solid Tumors (RECIST) version 1.1. Clinical and pathological characteristics of all patients included in our study were retrieved from the clinical records available. Inclusion criteria considered: (a) Eastern Cooperative Oncology Group (ECOG) Performance Status (PS) of ≤ 2; (b) patients with histologically‐ or cytologically documented NSCLC with unresectable stage IIIB‐C or Stage IV Disease (according to version 8 of IASLC TNM) who were treatment‐naïve and eligible for first‐line active systemic treatment according to clinical practice (osimertinib [osi], alectinib, pembrolizumab [pembro] or platinum chemotherapy [CT]‐based treatments [CT +/− pembro]). Exclusion criteria included (a) patients with other malignant tumors; (b) patients with ECOG PS ≥ 3; (c) patients who received prior systemic oncological treatment; (d) patients with mental illness prohibiting informed consent. Of note, to reduce the interference with EVs isolation and kinetics, only patients not affected by other medical conditions and not receiving other concomitant medications were finally considered for study analysis. The study was conducted following the Declaration of Helsinki, and the protocol was approved by The Ethics Committee Palermo I (AIFA code CE 150109). Again, experiments were undertaken with the understanding and written consent of each subject.

### 2.3 Tumor tissue collection, nucleic acids extraction and molecular analysis

Tumor tissue was obtained by systematic biopsy at baseline and stored as a formalin‐fixed paraffin‐embedded (FFPE) sample at the Department “G. D'Alessandro”, Pathology Institute, University of Palermo and at other referring Pathology Units. All FFPE and plasma samples were analyzed in the Laboratory of Molecular Oncology at the “Regional Reference Center for the prevention, diagnosis and treatment of rare heredo‐familial cancers of adults Medical Oncology” (Medical Oncology Unit, A.O.U.P. “P. Giaccone”, University Hospital of Palermo). The mutational status for the detection of oncogene addiction was tested on the thickness of 10 μm tissue sections obtained from biopsy at baseline or resected tumor tissue stored as FFPE samples. PD‐L1 immunohistochemistry (IHC) was carried out on 4‐μm sections of FFPE tumor tissue samples using Dako PD‐L1 IHC 28‐8 PharmaDx (Agilent, Santa Clara, CA, USA) and evaluated by a trained pathologist according to the tumor proportion score (TPS), defined as the percentage of positive viable tumor cells among all viable tumor cells evaluated. DNA and RNA nucleic acids were extracted from six 10 μm thickness FFPE tissue sections with an adequate percentage of neoplastic cells ≥ 20%. The genomic DNA and RNA were extracted from FFPE tissue using the QIAmp FFPE Tissue Kit and RNeasy FFPE kit (Qiagen, Hilden, Germany), respectively, and quantified in terms of ng·μL^−1^ using Qubit™ dsDNA HS Assay Kit and Qubit™ RNA HS Assay Kit (ThermoFisher Scientific, Foster City, CA, USA), respectively. According to clinical practice, 10 ng of both DNA and RNA including *EGFR*, *BRAF*, *KRAS*, *ALK*, ROS1 genes alterations, *MET* amplification, and eventually the gene fusion transcripts were tested using the Oncomine™ Focus DNA/RNA panel. Moreover, the Oncomine™ RNA assay offered the opportunity to evaluate the 5′/3′ imbalance ratio at the *ALK*, *ROS1*, *RET* and *NTRK* genes as a fusion signature independently by the unknown partner. Libraries were quantified by Ion Library TaqMan™ quantification kit on QuantStudio7 Pro Real‐Time PCR System (Applied Biosystem, Waltham, MA, USA) using design & analysis Software v2.4.3. The analytical sensitivity of the assay achieved at an allelic frequency ≥ 5% was 100%, but the performance of every single run was referred to the data. The data were tested on an amplicon‐based sequencing platform Ion Torrent S5™ System (Thermo Fisher Scientific, Waltham, MA, USA). The Oncomine Focus‐520‐w.30‐DNA‐Single Sample and the Oncomine Focus‐520‐w.30‐Fusions‐Single Sample represented the workflow applied for the analysis of DNA and RNA samples. To test the reliability of data for DNA sequencing, we complied with the following thresholds: mapped read > 300 000.00, mean read length > 75 bp, uniformity > 90%, and mean raw accuracy > 99%. For RNA sequencing, we considered an analysis with mapped read > 50 000, mean read length > 60 bp, and expression controls detected ≥ 3 out of 5. The data of DNA sequencing were analyzed with Ion Torrent torrentsuite™ (TS, version 5.18) processing the plug‐in of Coverage Analysis and Variant Caller. integrative genomics viewer (IGV v2.4.1, https://igv.org/) [25] was used to visibly evaluate the alignments of sequences. Pathogenetic changes in DNA and RNA sequences with the related percentage of allelic frequency were annotated, only for DNA analysis, by ion reporter Software v5.18 (Thermo Fisher Scientific, Waltham, MA, USA) applying the filter chains Oncomine variants for default use and default fusion view 5.18 (Thermo Fisher Scientific, Waltham, MA, USA), respectively, and were described using the Human Genome Variation Society (HGVS) standard nomenclature. To confirm the data of common pathogenic variants or the cases of poor quality and quantity DNA, 15–30 ng of DNA was amplified using EasyPGX ready *EGFR*/*BRAF*/*KRAS* kit with a LOD of 5%. After about 2 h run, the data obtained on Real‐Time EasyPGX System (Diatech Pharmacogenetic, Jesi AN, Italy) were analyzed using easypgx Analysis Software v4.0.10 (Diatech Pharmacogenetics srl, Jesi, Italy).

### 2.4 Blood samples and plasma separation

Blood samples (5 mL) were collected into K_2_ EDTA tubes (BD Vacutainer®) early in the morning with fasting required. Blood samples from included patients were collected at baseline before the first drug administration and at each instrumental disease re‐evaluation during the treatment course according to clinical practice. As previously described [26], blood specimens were immediately processed for plasma collection and centrifuged twice. The first refrigerated centrifugation was performed using low force for 10 min at 1200 ***g*** to retain the vast majority of EVs and exclude cellular material. The second centrifugation was carried out for 10 min at 16 000 ***g*** to completely remove some of the large vesicles including cellular fragments and cell debris. Sample processing was carried out within 15 min of blood collection. An aliquot (2 mL) of collected plasma was immediately processed for isolating EVs, whereas other aliquots were frozen at −80 °C for subsequent biophysical or biological analysis. Further, to remove the cryoprecipitates, the appropriately thawed plasma aliquot was centrifugated for 5 min at 3000 ***g*** and 4 °C before the EV isolation steps.

### 2.5 Extracellular vesicles isolation

According to the most recent ISEV guidelines [27], following serial centrifugation steps, EVs were isolated using a membrane affinity‐based method [9, 28]. Briefly, EVs were isolated using the exoEasy Maxi Kit (Qiagen), based on a membrane affinity purification method, according to the manufacturer's protocol. Specifically, non‐vesicular proteins along with organic polymers and other impurities were washed out during a column‐based centrifugation procedure using such a membrane affinity method.

### 2.6 Extracellular vesicles characterization by western blot

Extracellular vesicles were analyzed to detect specific proteins well‐known as markers of the EV population. Twenty microgram of EVs were resolved by Novex Bis‐Tris SDS‐acrylamide gels ( Thermo Fisher Scientific, Waltham, MA, USA) in reducing conditions and with heating. After the electrophoresis, proteins were transferred to nitrocellulose membranes (GE Healthcare Life Sciences, Boston, MA, USA) and non‐specific binding sites were blocked by incubating membranes in a blocking solution: 10% non‐fat dry milk (Sigma‐Aldrich, St. Louis, MO, USA) in Tris‐Buffer Saline, 0.1% Tween (TBS‐T), for 60 min, at room temperature. Then, membranes were incubated overnight at 4 °C with monoclonal anti‐TSG‐101 (dilution 1 : 500; sc‐7964) and ALIX (dilution 1 : 500; sc‐53540) antibodies; anti‐CD63 (clone MX49; dilution 1 : 200; sc‐5275) and anti‐CD81 (clone: B11; dilution 1 : 1000; sc‐166029); after three washes in TBS‐T, the membranes were incubated with specific secondary HRP‐antibodies (dilution 1 : 10 000) for 1 h (Santa Cruz Biotechnology Inc, Dallas, TX, USA). Chemiluminescence was detected using the Chemidoc system (BioRad, Hercules, CA, USA).

### 2.7 Extracellular vesicles characterization by dynamic light scattering

Dynamic light scattering assay was used to evaluate the biophysical biomarker of an aliquot of isolated EVs from patients using PBS and a commercial elution buffer (XE; Qiagen) as negative controls. Each isolated EV sample was poured directly into a quartz cuvette and centrifugated at 1000 ***g*** for 10 min and 4 °C to remove any dust particles. Subsequently, isolated EVs were placed at 20 °C in a thermostatic cell compartment of a BI200‐SM goniometer (Brookhaven Instruments, Nashua, NH, USA) equipped with a He‐Ne laser (JDS Uniphase 1136P) at 633 nm and a single pixel photon counting module (C11202‐050; Hamamatsu, Hamamatsu City, Japan). The scattered light intensity and its time autocorrelation function *g* ^2^(*t*) were measured simultaneously at 90° by using a BI‐9000 correlator (Brookhaven Instruments). Absolute values for scattered intensity (excess Rayleigh ratio at 90°, R90) were obtained by normalization to toluene and subtraction of the buffer signal [29]. R90 is proportional to the particle number concentration *N*, the squared weight‐averaged mass (Mw)_2_, and the form factor *P*(*q*); therefore, in the case of particles with the same size and shape, it can be considered as a rough esteem of the vesicle amount [30]. The autocorrelation functions were fitted by a two‐component Schultz distribution for the diffusion coefficient *D* [31, 32]. Then, the intensity‐weighted distribution of hydrodynamic radii *D*_h_ is determined by using the Stokes‐Einstein relation *D* = (*k*_B_ *T*)/(3πη*D*_h_), where *k*_B_ is the Boltzmann constant, *T* is the temperature, and η is the medium viscosity, that is assumed to be the same as PBS. Indeed, a DLS measurement was performed both on a vesicle sample in buffer XE and the same sample in PBS. This buffer exchange was performed by HPLC‐SEC run on a Sepharose CL‐2B column by recovering the void volume fraction to eliminate the small‐sized particles due to buffer XE. The first component of the distribution (not shown) amounts to < 5% of the signal and refers to small‐size particles (< 20 nm) present in the samples. By considering the second component of the distribution, which is related to vesicles, one measures the average and the normalized variance, corresponding to the z‐averaged hydrodynamic diameter (Dz) and the polydispersity index (PDI) of vesicle distribution, respectively [33].

### 2.8 Extracellular vesicles quantification by Bradford assay

Extracellular vesicle protein levels defined as cell‐free EV protein content (cfEV) were determined using BA. In brief, 10 μL of EVs resuspended in PBS were added to 200 μL of Coomassie Protein Assay Reagent (Pierce, Rockford, IL, USA). The absorbance at 595 nm was measured using the spectrophotometer (SPECTROstar nano; BMG LABtech, Ortenberg, Germany). The protein concentration was calculated using a standard curve of a dilution series of bovine serum albumin (BSA; Merck, Darmstadt, Germany) according to standard protocols from our group [34].

### 2.9 Statistical considerations

Descriptive statistics were used to analyze demographic and clinical data. According to radiologic response, efficacy was defined as responsive (complete response [CR] or partial response [PR]) or non‐responsive disease (stable disease [SD] or progressive disease [PD]), based on the standard RECIST 1.1 criteria. A paired Wilcoxon test was used to compare the mean EV plasma levels before and after the treatment course. Regarding cfEV, we dichotomized values as ≥ and < 20% indicating the change from baseline cfEV to higher and lower levels, respectively, after the initiation of treatment. The Kaplan–Meier method was used to perform survival analysis providing median values and their 95% confidence interval (CI) with *P*‐values, while the log‐rank test was selected for comparisons. Univariable and multivariable analyses were performed using the Cox proportional hazards regression models. The multivariable model included as covariates all pretreatment clinically meaningful parameters and/or statistically significant *P*‐values at univariable analysis. A logistic regression model was performed to evaluate the relationship between one or more independent variables with a dependent one. Progression‐free survival (PFS) was calculated from the date of study inclusion to the first evidence of disease progression or death from any cause or censored at the most recent follow‐up. A *P*‐value < 0.05 was used as the threshold for statistical significance. All the statistical analyses were performed using the spss statistics software, version 20 (IBM, Armonk, NY, USA).

## 3 Results

With a median follow‐up of 34.2 months (range: 24.8–42.0 months), among 87 patients meeting the eligibility criteria, 27 consecutive patients with advanced NSCLC and no other medical conditions or concomitant medications were prospectively included. Briefly, a total of 135 liquid biopsy samples were collected isolating EVs from 27 patients at baseline with paired available plasma samples at disease radiologic re‐evaluation. To understand if EV biophysical properties can be used as complementary tools to assess cancer response in the clinical setting of advanced NSCLC, we further categorized the overall population by specific treatment subgroups (osi, pembro, and CT +/− pembro). Clinicopathological characteristics are summarized in (Table S1).

### 3.1 Plasma EVs characterization and frequencies over treatment

**Fig. 2:**
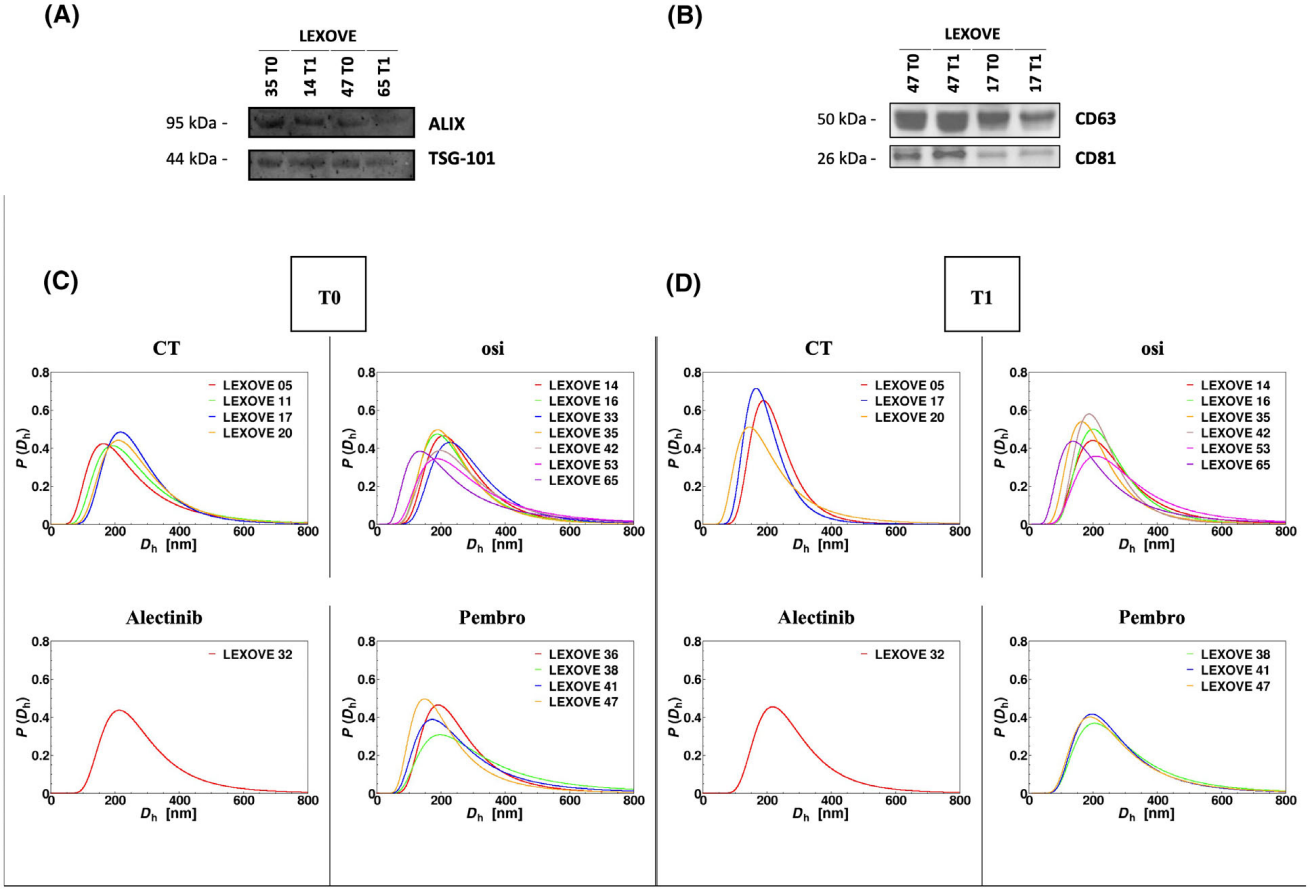
Extracellular vesicles (EVs) characterization: Western blot showing positive EV staining for ALIX and TSG‐101 (A), CD63 and CD81 (B) both at baseline (T0) and restaging (T1) timepoint; Dynamic Light Scattering: *P*(*D*_h_) curves represent the size distribution of EVs isolated at T0 (C) and T1 (D) from plasma of treatment‐naïve patients with advanced NSCLC undergoing first‐line systemic treatments. CT, chemotherapy; Dz, z‐averaged hydrodynamic diameter; osi; osimertinib; *P*(*D*_h_), distribution function of the hydrodynamic diameter; pembro, pembrolizumab.

**Fig. 3:**
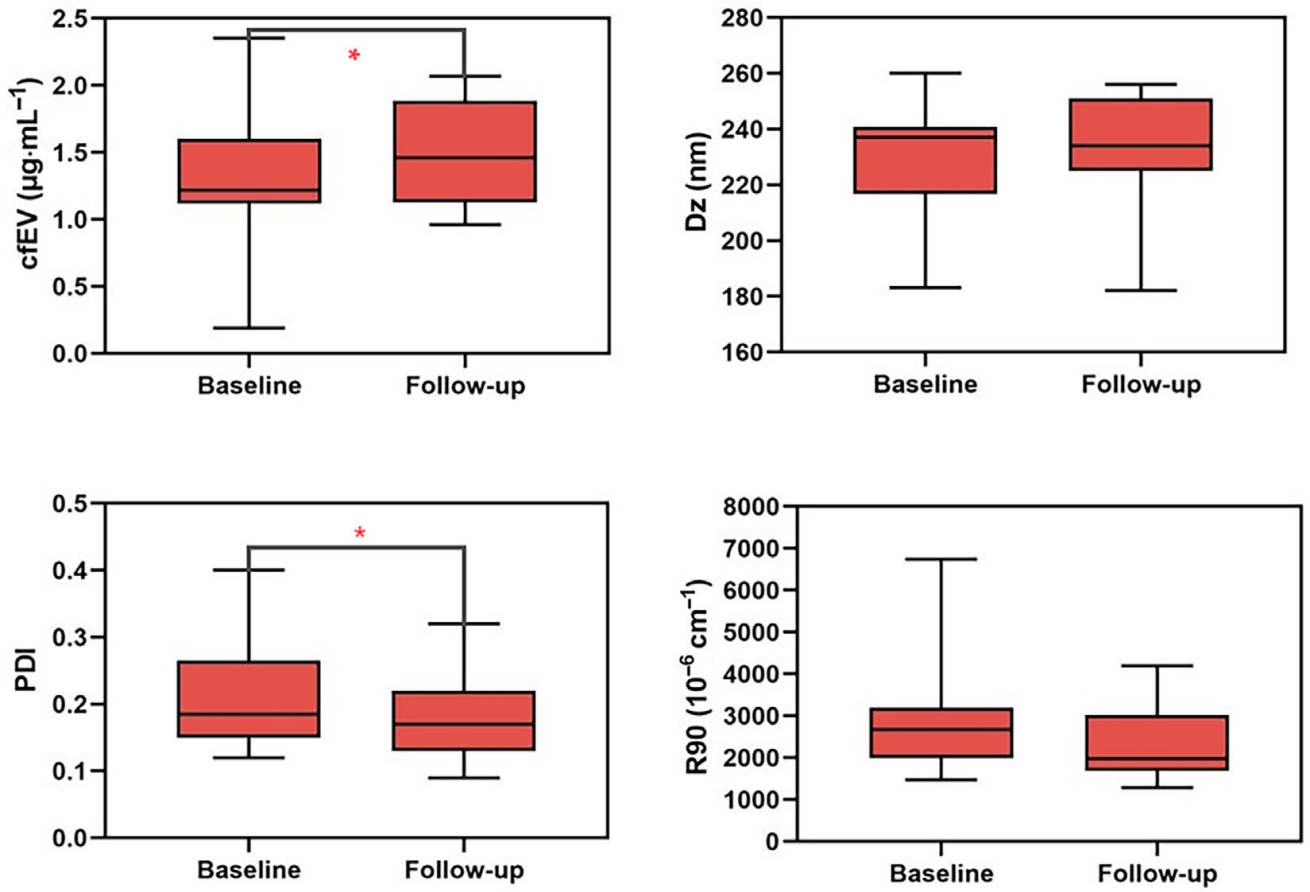
Box‐and‐whisker plots showing different median levels of plasma extracellular vesicles at baseline (T0) and after 12 weeks follow‐up (T1) according to Bradford and Dynamic Light Scattering analyses; boxes indicate median and IQR (25th and 75th percentiles), whiskers are plotted using the quartile ± 1.5 × IQR convention and asterisks (*) denote statistically significant values (calculated with *t*‐Student test). cfEV, cell‐free extracellular vesicle protein content; Dz, z‐averaged hydrodynamic diameter; IQR, interquartile range; PDI, polydispersity index; R90, excess Rayleigh ratio at 90°.

**Fig. 4:**
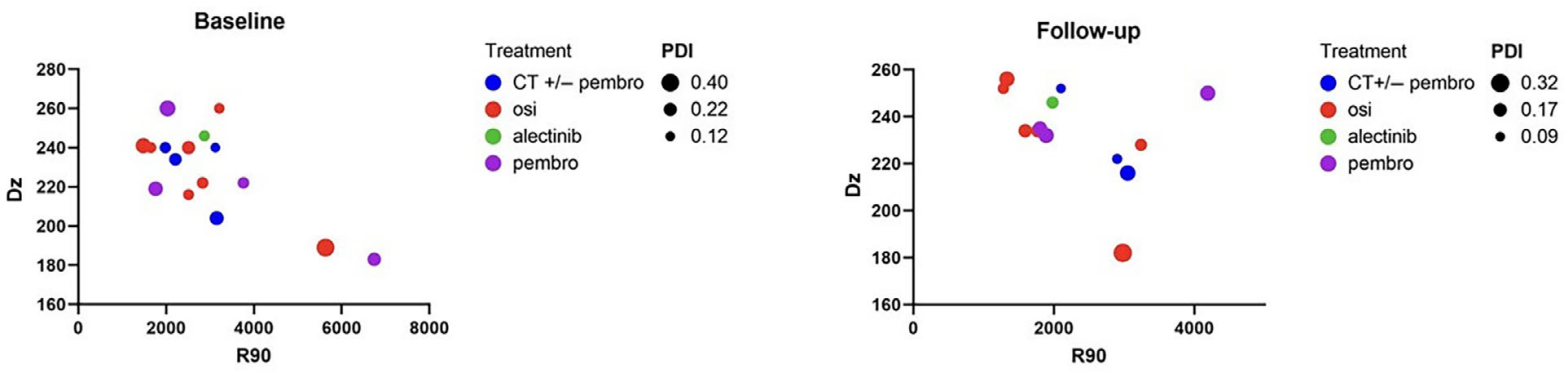
Dynamic light scattering showing amount (R90), size (Dz) and distribution (PDI) of extracellular vesicles at baseline (T0) and after 12 weeks follow‐up (T1). CT, chemotherapy; Dz, z‐averaged hydrodynamic diameter; osi; osimertinib; PDI, polydispersity index; pembro, pembrolizumab; R90, Rayleigh ratio.

Specific markers, protein content, size, and amount of plasma EVs were examined (Fig. 2). Isolated EVs were characterized by western blot (WB) revealing conventional cytosolic EV markers such as Alix and TSG‐101 (Fig. 2A) together with multi‐pass transmembrane proteins such as tetraspanins CD63 and CD81 (Fig. 2B). Four representative samples (one for each specific treatment subgroup at T0 and T1) are shown in Fig. 2A. The assessment of EV‐associated category 1 and 2 proteins confirmed the presence of EV features that did not show any on‐treatment intraindividual differences between baseline and disease restaging (Fig. 2A,B). Morphological analysis of EVs was performed by DLS taking into consideration R90, Dz, and PDI values both at T0 and T1 (Figs 3 and 4). DLS showed a Dz value ranging from 183 to 260 nm with an average size of 228.5 and 233.7 nm at baseline and restaging, respectively, without any on‐treatment significant differences in diameters (*P* = 0.24) (Figs 2C,D and 3, Table S2). An average R90 value of 2964.75 and 2315.54 × 10^−6^ cm^−1^ was found at T0 and T1 with no significant changes regarding the amount of EVs over therapy (*P* = 0.21) (Fig. 3, Table S2). We observed an average PDI value of 0.21 and 0.18 at baseline and restaging, showing statistically significant differences in the width of distribution size of EVs over treatment (*P* = 0.03) (Fig. 3, Table S2). BA of plasma EVs showed a mean protein value of purified cfEVs of 1.26 and 1.49 μg·mL^−1^ at baseline and restaging, respectively, resulting in statistically significant changes during the treatment course (*P* = 0.02) (Fig. 3, Table S2).

### 3.2 Association of plasma EVs dynamics with survival according to treatment subgroups

**Fig. 5:**
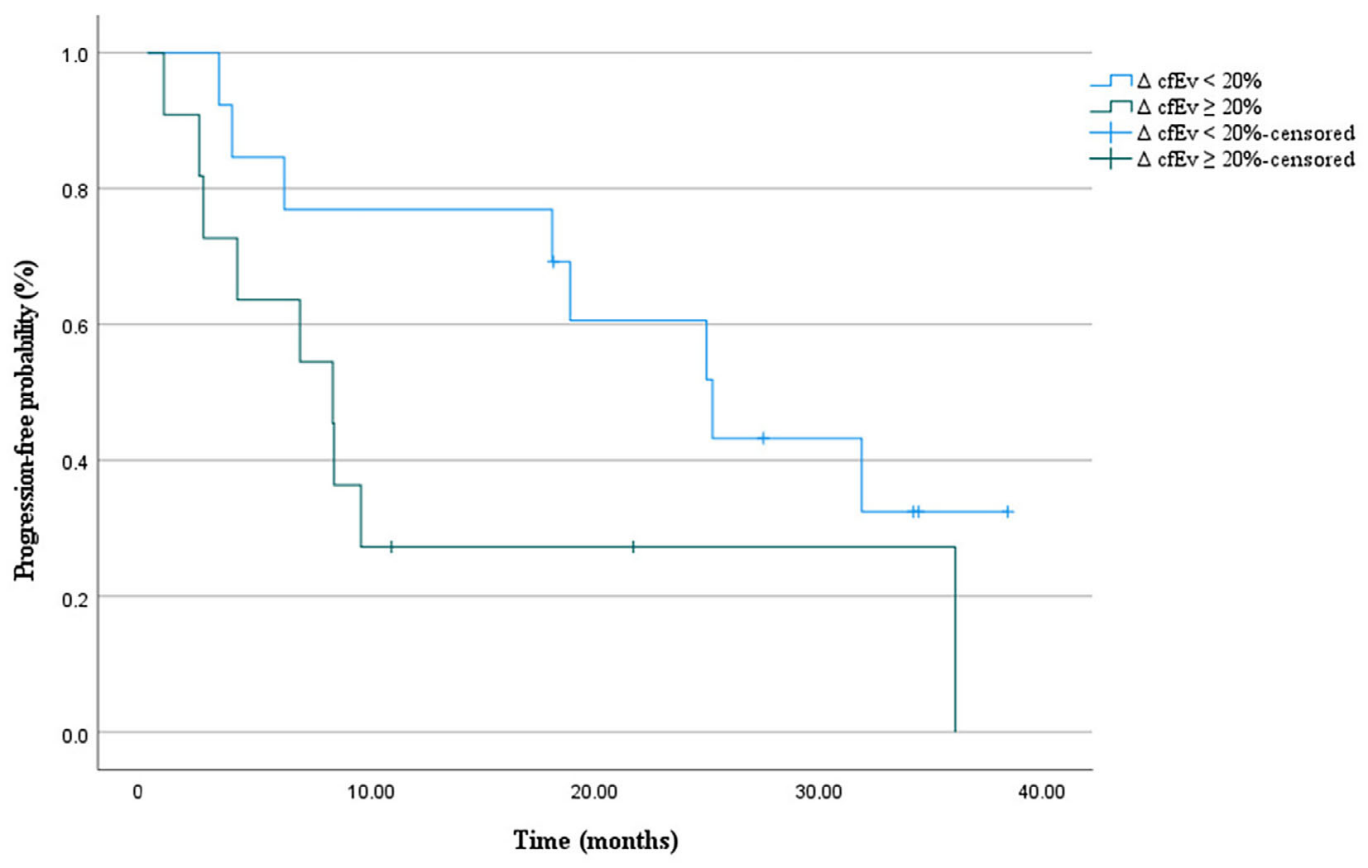
Kaplan–Meier analysis of progression‐free survival according to Δ cfEV in the overall cohort population. Δ cfEV, cell‐free extracellular vesicle protein levels from baseline to disease restaging.

**Fig. 6:**
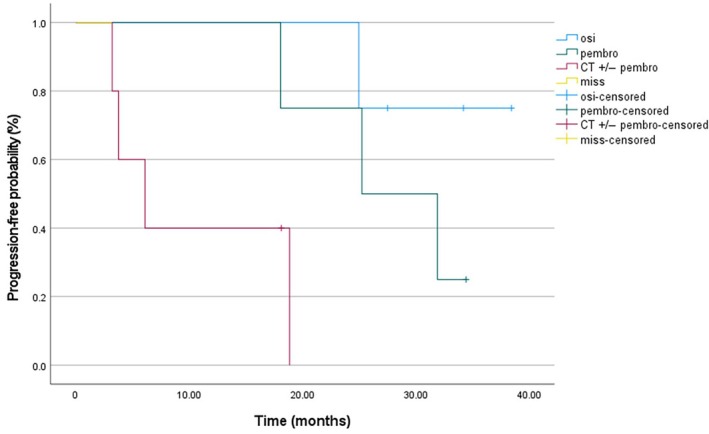
Kaplan–Meier analysis of PFS according to treatment subgroups in patients with Δ cfEV decrease. CT, platinum‐based chemotherapy; osi, osimertinib; pembro, pembrolizumab; Δ cfEV, cell‐free extracellular vesicle protein levels from baseline to disease restaging.

As previously evaluated for cfDNA kinetics [26], we evaluated the dynamics of cfEV in the overall cohort population and across treatment subgroups according to available plasma samples, detecting a 20% median increase of cfEV as the cut‐off point for analyses of patients receiving osi, pembro or CT +/− pembro. Overall, 13 patients with Δ cfEV decrease had clinically improved mPFS (25.2 months, 95% CI: 14.9–35.5) when compared to 11 patients with Δ cfEV increase (8.3 months, 95% CI: 3.6–12.9), trending to the formal significance (*P* = 0.07) (Fig. 5). Namely, dealing with treatment subgroups, patients receiving single‐agent pembro with decreasing Δ cfEV levels presented with a statistically improved mPFS (25.2 months, 95% CI: 11.7–38.8) compared to those patients with increasing Δ cfEV (6.8 months, 95% CI: 0–6.8) (*P* = 0.04). To the contrary, no differences in mPFS according to Δ cfEV were observed in the CT +/− pembro group (6.1 [95% CI: 1.1–11.1] vs. 8.3 months [95% CI: 7.7–10.1]; *P* = 0.9). Intriguingly, EGFR‐positive patients receiving osi with Δ cfEV decrease tended to experience longer mPFS (35.1 months, 95% CI: 14.9–35.5) as compared to patients with increasing Δ cfEV values (20.8 months, 95% CI: 11.2–30.4) (*P* = 0.06). Figure 6 depicts the Kaplan–Meier plot of PFS according to treatment subgroups in patients with Δ cfEV decrease.

### 3.3 Univariable and multivariable associations between potential risk factors and extracellular vesicles dynamics

Different clinical variables were evaluated as potential independent risk factors for PFS or cfEV dynamics using Cox proportional hazards regression analysis. The tested variables were gender, age, smoking status, histology, treatment, tissue PD‐L1 expression levels, and cfEV kinetics. Finally, the multivariable analyses suggested ECOG PS of 2 as the only independent risk factor for survival whereas none of the tested clinical parameters resulted in significantly affecting the cfEV dynamics during the treatment course (Tables S3 and S4).

### 3.4 Longitudinal monitoring by liquid biopsy data

**Fig. 7:**
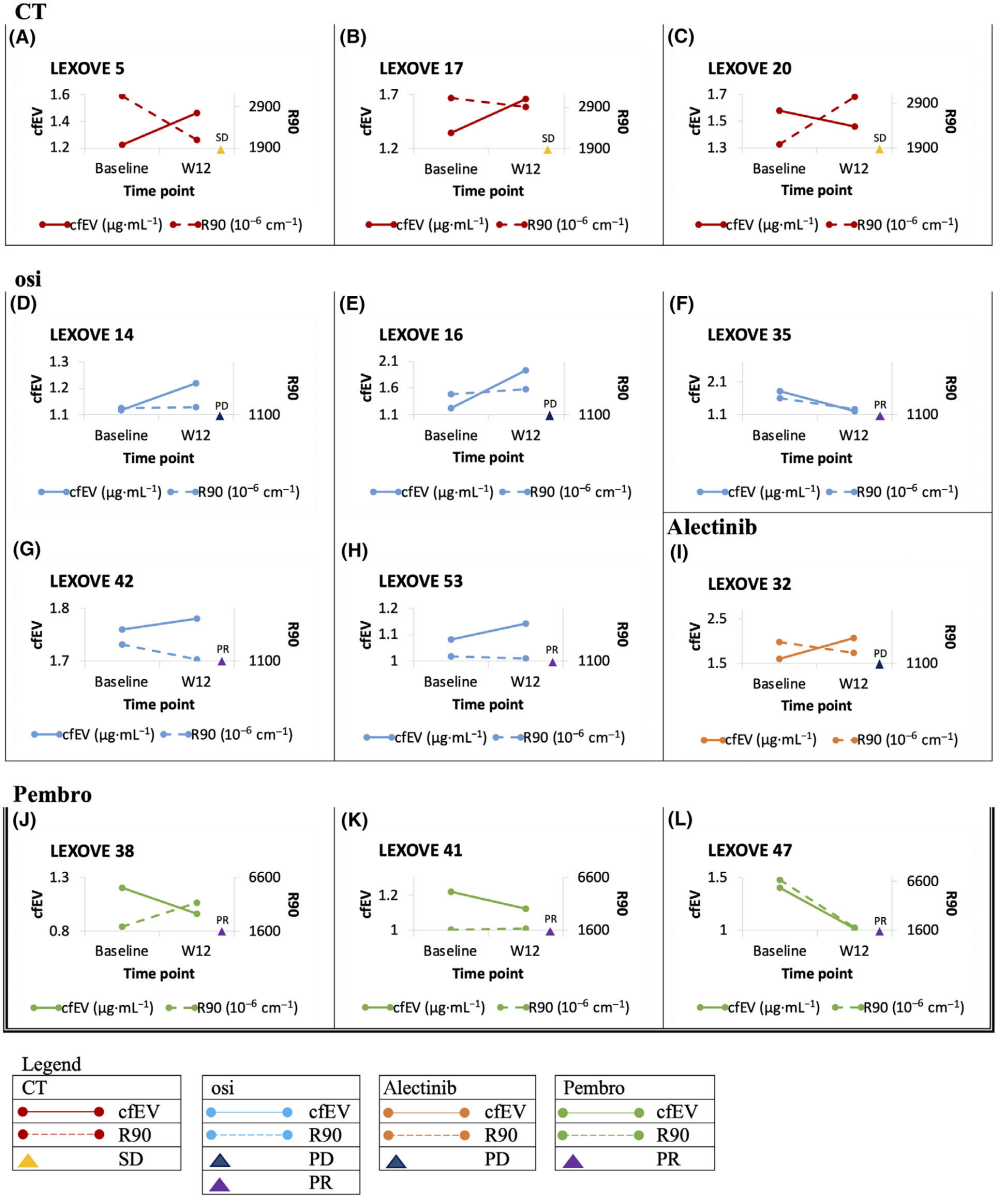
Graphical representation of the longitudinal monitoring of mNSCLC patients (listed as A–L) according to the dynamics of amount (R90) and protein levels (cfEV) of extracellular vesicles (EVs) among different treatment subgroups. cfEV, cell‐free extracellular vesicle protein levels; CT, chemotherapy; osi; osimertinib; PD, progressive disease; pembro, pembrolizumab; PR, progressive response; R90, the Rayleigh scattering values; SD, stable disease; W12, first disease restaging at 12 weeks.

Finally, focusing on patients with sufficient biospecimens for EV analysis and at least two clinical follow‐ups for therapeutic assessment, here we report the graphical representation of the results of the longitudinal monitoring by liquid biopsy (R90 and cfEV) (Fig. 7). Interestingly, dealing with the EGFR‐positive subgroup, one patient with R90 increasing value faced a rapidly progressive disease (Fig. 7D) whereas the other four patients with R90 decreasing values are still responding to treatment (Fig. 7E–H, Table S2).

## 4 Discussion

Despite emerging personalized treatments have dramatically changed the clinical practice of NSCLC, unfortunately, the prognosis of such patients with advanced disease remains poor with treatment response being regrettably confined to specific subsets of patients [35]. In the precision oncology era, research on biomarker‐driven treatment has been rapidly evolving with different liquid biopsy analytes being intensively investigated for diagnosis and prognostication in the most common solid tumors [14]. Over the last decade, a growing body of emerging preclinical data supported the investigation of EVs as a compelling source of cancer biomarkers leading to differing retrospective series that evaluated the sampling of plasma EVs as a promising approach to dynamically track the real‐time cancer behavior [36, 37]. However, the significance of EV contents in *ex‐vivo* samples from prospective clinical cohort trials remains poorly understood. In this fascinating scenario, we designed a prospective biomarker trial to evaluate the dynamics of plasma EVs profile as prognostic and/or predictive biomarkers in the first‐line clinical setting of NSCLC.

First, we demonstrated the feasibility of isolating and characterizing EVs from the plasma of treatment‐naïve patients in a real‐life setting of advanced NSCLC. To improve the separation efficiency and enrichment during the isolation steps, we used serial centrifugation steps followed by a membrane affinity‐based method that could yield purified vesicle fraction by largely removing co‐isolating structures (such as protein complexes or lipoproteins, especially abundant in plasma), thus confirming the improved performance of combined isolation methods when dealing with clinical samples [38, 39]. EV characterization, through the evaluation of well‐known protein markers, was assessed by WB. EV size was analyzed by integrating DLS, a Brownian motion‐based technique that evaluated the integrity and purity of EVs, taking into consideration R90, Dz and PDI for the estimation of the amount, diameter, and size distribution of EVs, respectively. EVs were further characterized using BA, a cheap and easy‐to‐use colorimetric assay that informatively quantified the protein amount of purified EVs in our cohort. This was consistent with previous reports that however used dissimilar techniques such as transmission electron microscopy and flow cytometry [40]. Finally, considering a PDI value not > 0.4 in the entire cohort, we detected a homogenous population of vesicles within monodispersed plasma samples [41]. Noteworthy, it should be considered that the biological response of EV cargo could be affected by its dispersion state and solubility in plasma. In our study, compared to microscopy techniques, DLS provided additional data of paramount significance on the biodistribution of nanoparticle properties. An added advantage is that DLS is not time‐consuming, not as technically challenging, and does not result in any sample loss, making it a valuable approach for the quantification of plasma EV levels and the detection of residual small‐sized co‐isolates, especially when dealing with scant and precious patients' plasma samples [42]. These data support the use of DLS as a valid technique for characterizing *in vivo* EVs with a low limit of detection from the plasma of patients with advanced NSCLC, confirming previous findings from other solid tumors [43, 44]. In addition, we leveraged the combination of DLS with BA to improve sample characterization and further investigate EV protein content, demonstrating that this approach in NSCLC patients allowed a quantitative and high‐throughput characterization of purified EVs even in cases of limited sample volume.

Then, we asked whether the dynamics of plasma EVs from baseline to first radiologic restaging could unveil patients' survival according to specific treatment subgroups. We observed that the level of plasma EV proteins and the width of EV distribution were significantly affected over time by the treatment course whereas EV diameters did not show any statistically significant on‐treatment variations. Interestingly, in the overall cohort, we observed that dynamic EV protein changes were associated with sustained treatment responses and clinically improved survival since responders exhibited decreasing levels of cfEV compared to a significant increase in non‐responders. Strikingly, this was clinically prominent in those patients receiving single‐agent pembro or osi, compared to those patients undergoing chemotherapy +/− pembro. In the pembro subgroup, the Δ cfEV was significantly associated with survival (*P* = 0.04) whereas in the osi arm the threshold for statistical significance was not formally reached (*P* = 0.06). Considering such survival advantage in patients presenting with cfEV decreasing levels, it could be argued that, especially in the EGFR‐positive population, EV dynamics can mediate the EV translocation of functionally active receptors and ligands in the cell–cell communication process with local and distant cells, as emerged over the past decade [45]. Importantly, we proved that cfEV kinetics was not affected by any baseline patients' clinical characteristics while, in line with other evidence, the multivariable analysis identified only ECOG PS as a potentially interfering risk factor associated with worse survival in the overall cohort [46].

The limitations of this study include the non‐randomized design, the small sample size, the heterogeneity of clinical‐pathological characteristics, and the lack of results in terms of overall survival. As regards the limited sample size, it should be noted that, to strictly limit any selection or sample analysis biases affecting the outcome of EV experiments, according to the latest ISEV guidelines we only considered and tested fasting plasma from a real‐life consecutive series of advanced NSCLC patients presenting with no other co‐existing medical conditions or concomitant medications [27]. Moreover, to test for the variability of EV‐based features and to further validate our results, we analyzed matched intraindividual plasma samples with repeated plasma samples serving as controls not eventually affected by individual differences. Considering the immature follow‐up and the absence of enough death events, here we did not present any results of EV dynamics in terms of overall survival that would however have been affected by subsequent treatment lines. Besides, a significant constraint could regard the use of DLS that could not characterize the specific phenotype of the isolated EVs, albeit resulting to be extremely useful when characterizing precious plasma samples. In this regard, despite the need for optimizing its analytical validity, integrating the evaluation of plasma stable EVs could contribute to improving the performance of liquid biopsy assays for treatment monitoring and stratification, avoiding biased and inaccurate results that would derive from time‐consuming and costly omics analyses carried out on often degraded and contaminated cfDNA. This proof‐of‐concept study provided preliminary evidence on the potential role of plasma EVs as circulating biomarkers generating a study hypothesis that should be adequately addressed by larger prospective trials for obtaining accurate, reliable, and fast biomarkers of treatment response, mostly in those patients who are candidates to receive first‐line single‐agent pembro or osi.

## 5 Conclusions

The results of this study showcased the feasibility of the serial on‐treatment monitoring of plasma EVs in the first‐line setting of NSCLC, providing an added value in the real‐time monitoring of treatment response which is more prominent in those patients receiving first‐line single‐agent pembro or osi. Besides the efficacy of the isolation methods and the purity of the retrieved EV fraction, some practical considerations such as costs, hands‐on time, and the total duration of isolating procedures should be pondered [47]. The increased amount of circulating EVs (R90) and the higher level of associated proteins (cfEV) might better reflect the biology of NSCLC. This evidence, together with the increased stability in plasma, warrants larger controlled studies to explore EVs as novel promising liquid biopsy biomarkers even for early cancer detection, as recently outlined [48]. Despite contemporary EV research extensively relies on a wide range of biochemical and physical analysis methodologies, it is essential to delve deeper into *in vivo* samples and validate such differing EV‐based approaches in the clinic for diagnostic and therapeutic purposes.

## Conflict of interest

The authors declare no conflict of interest.

## Author contributions

VG, VB, and NB contributed to conceptualization; VG, NB, ST, and APC contributed to methodology; VG and AG contributed to software; VG, VB, NB, UM, and DS contributed to validation; VG, NB, MM, SR, APC, and AG contributed to formal analysis; VG, NB, ST, AG, MM, and SR contributed to investigation; VG, NB, VB, ST, SR, MM, APC, and AR contributed to resources; MB, TDBR, FP, PP, and GT contributed to data curation; VG, NB, MM, SR, and AG contributed to writing—original draft preparation; LI, GB, VB, GT, UM, AR, and DS contributed to writing—review and editing; VG, NB, and ST contributed to visualization; LI, GB, GT, VB, UM, AR, DS, and AG contributed to supervision; VG, NB, VB, AR, and DS contributed to project administration. All authors have read and agreed to the published version of the manuscript.

## Supporting information


**Table S1.** Baseline characteristics of the enrolled treatment‐naïve patients with advanced NSCLC.
**Table S2.** Extracellular vesicles (EV) dynamics according to Bradford Assay and Dynamic Light Scattering analyses at baseline and after twelve weeks follow‐up.
**Table S3.** Univariable and multivariable analysis for progression‐free survival (PFS) in enrolled patients.
**Table S4.** Association between clinical characteristics and circulating extracellular vesicle protein level (cfEV) dynamics in enrolled patients.

## Data Availability

Data could be available upon reasonable request to the authors.
