# Supplementary material for: On‐treatment dynamics of circulating extracellular vesicles in the first‐line setting of patients with advanced non‐small cell lung cancer: the LEXOVE prospective study
Source: Mol Oncol. 2025 Jan 9;19(5):1422–35. doi: 10.1002/1878-0261.13737 (PMC12077285; doi:10.1002/1878-0261.13737)
Supplement: Supplementary file 1 — Table S1. Baseline characteristics of the enrolled treatment‐naïve patients with advanced NSCLC. Table S2. Extracellular vesicles (EV) dynamics according to Bradford Assay and Dynamic Light Scattering analyses at baseline and after twelve weeks follow‐up. Table S3. Univariable and multivariable analysis for progression‐free survival (PFS) in enrolled patients. Table S4. Association between clinical characteristics and circulating extracellular vesicle protein level (cfEV) dynamics in enrolled patients. [file MOL2-19-1422-s001.docx]

**Supplementary Tables**

**Supplementary table S1.** Baseline characteristics of the enrolled treatment-naïve patients with advanced NSCLC.

|  | | **Population** | |
| --- | --- | --- | --- |
| **Characteristics** | | **Number of patients (%)** |  |
| **Gender** | Men | 18 (66.7%) |  |
|  | Women | 9 (33.3%) |  |
| **Age (years)** | Mean ± Standard deviation | 70 +/- 8.6 |  |
|  | <65 | 6 (22.2%) |  |
|  | ≥65 | 21 (77.8%) |  |
| **Smoking habits** | Never smoker | 4 (14.8%) |  |
|  | Current or former smoker | 23 (85.2 %) |  |
| **ECOG PS** | 0-1 | 14 (51.8%) |  |
|  | 2 | 13 (48.1%) |  |
| **Histological subtype** | Non-SCC | 20 (74.1%) |  |
|  | SCC | 7 (25.9%) |  |
| **Molecular diagnostics** | *EGFR* | 8 (29,6%) |  |
|  | *ALK* | 3 (11.1%) |  |
|  | *ROS-1* | 1 (3.7%) |  |
|  | wild-type | 15 (55.6%) |  |
| **Tissue PD-L1 (TPS)** | Negative (<1%) | 7 (25.9%) |  |
|  | Low expression (1-49%) | 12 (44.4%) |  |
|  | High expression (≥50%) | 7 (25.9%) |  |
| **Site of disease** | Intra-thoracic | 10 (37.0%) |  |
|  | Extra-thoracic | 14 (51.9%) |  |
| **Treatment** | pembrolizumab | 6 (22.2%) |  |
|  | CT +/- pembrolizumab | 9 (33.3%) |  |
|  | alectinib | 4 (14.8%) |  |
|  | osimertinib | 8 (29.6%) |  |
| **Radiologic response** | Partial response | 14 (51.9%) |  |
|  | Stable disease | 3 (11.1%) |  |
|  | Progressive disease | 10 (37%) |  |
| **Progression** | Yes | 10 (37%) |  |
|  | No | 26 (63%) |  |
| **PFS (months)** | Median (range) | 9.53 (0 – 25.70) |  |
| **Death** | Yes | 20 (74.1%) |  |
|  | No | 7 (25.9%) |  |
| **OS (months)** | Median (range) | 23.33 (6.81 – 39.84) |  |

**Supplementary Table S2.** Extracellular vesicles (EV) dynamics according to Bradford Assay and Dynamic Light Scattering analyses at baseline and after twelve weeks follow-up. Abbreviations: cfEV, cell-free EV protein levels; R90, Rayleigh ratio; Dz, z-averaged hydrodynamic diameter; PDI, polydispersity index; W12, disease restaging after twelve weeks; NA, not available; CT, platinum chemotherapy; osi, osimertinib; pembro, pembrolizumab; SD, stable disease; PD, progressive disease; PR, partial response.

|  | |  | |  | **cfEV (µg/ml)** | | **R90 (****10-6 cm-1)** | | **Dz (nm)** | | **PDI** | |
| --- | --- | --- | --- | --- | --- | --- | --- | --- | --- | --- | --- | --- |
| **ID** | **Therapy** | | **Scan** | | **Baseline** | **W12** | **Baseline** | **W12** | **Baseline** | **W12** | **Baseline** | **W12** |
| LEXOVE5 | CT | | SD | | 1.22 | 1.46 | 3150 ± 125 | 2100 ± 105 | 204 ± 5 | 252 ± 10 | 0.25 ± 0.01 | 0.09 ± 0.01 |
| LEXOVE11 | CT | | PD | | 2.35 | NA | 2210 ± 150 | NA | 234 ± 15 | NA | 0.20 ± 0.01 | NA |
| LEXOVE14 | osi | | PD | | 1.12 | 1.22 | 1650 ± 95 | 1770 ± 75 | 240 ± 10 | 234 ± 10 | 0.15 ± 0.01 | 0.17 ± 0.01 |
| LEXOVE16 | osi | | PD | | 1.22 | 1.94 | 2830 ± 100 | 3240 ± 110 | 222 ± 5 | 228 ± 5 | 0.17 ± 0.01 | 0.14 ± 0.01 |
| LEXOVE17 | CT | | SD | | 1.34 | 1.66 | 3120 ± 100 | 2900 ± 75 | 240 ± 10 | 222 ± 5 | 0.12 ± 0.01 | 0.10 ± 0.01 |
| LEXOVE20 | CT | | SD | | 1.58 | 1.46 | 1980 ± 70 | 3050 ± 80 | 240 ± 10 | 216 ± 5 | 0.16 ± 0.01 | 0.23 ± 0.01 |
| LEXOVE32 | alectinib | | PD | | 1.6 | 2.06 | 2870 ± 100 | 1980 ± 90 | 246 ± 10 | 246 ± 10 | 0.15 ± 0.01 | 0.14 ± 0.01 |
| LEXOVE33 | osi | | PR | | 0.96 | 1.18 | 3210 ± 30 | NA | 260 ± 5 | NA | 0.14 ± 0.01 | NA |
| LEXOVE35 | osi | | PR | | 1.8 | 1.22 | 2510 ± 80 | 1590 ± 100 | 216 ± 5 | 234 ± 10 | 0.15 ± 0.01 | 0.17 ± 0.01 |
| LEXOVE36 | pembro | | PD | | 1.22 | NA | 3760 ± 150 | NA | 222 ± 10 | NA | 0.17 ± 0.01 | NA |
| LEXOVE38 | pembro | | PR | | 1.2 | 0.96 | 2030 ± 20 | 4190 ± 40 | 260 ± 5 | 250 ± 5 | 0.32 ± 0.01 | 0.22 ± 0.01 |
| LEXOVE41 | pembro | | PR | | 1.22 | 1.12 | 1758 ± 18 | 1802 ±18 | 219 ± 15 | 235 ± 7 | 0.27 ± 0.01 | 0.19 ± 0.01 |
| LEXOVE42 | osi | | PR | | 1.76 | 1.78 | 2510 ± 100 | 1280 ± 60 | 240 ± 10 | 252 ± 10 | 0.22 ± 0.01 | 0.12 ± 0.01 |
| LEXOVE47 | pembro | | PR | | 1.4 | 1.02 | 6740 ± 70 | 1890 ± 19 | 183 ± 5 | 232 ± 5 | 0.23 ± 0.01 | 0.22 ± 0.01 |
| LEXOVE53 | osi | | PR | | 1.08 | 1.14 | 1478 ±15 | 1330 ± 14 | 241 ± 5 | 256 ± 5 | 0.29 ± 0.01 | 0.22 ± 0.01 |
| LEXOVE65 | osi | | PR | | 0.8 | 1.06 | 5630 ± 60 | 2980 ± 30 | 189 ± 5 | 182 ± 10 | 0.40 ± 0.01 | 0.32 ± 0.01 |
| LEXOVE67 | pembro | | PR | | 1.6 | 1.67 | NA | NA | NA | NA | NA | NA |
| LEXOVE68 | CT+ pembro | | PR | | 1.74 | 1.87 | NA | NA | NA | NA | NA | NA |
| LEXOVE69 | IO | | PR | | 1.14 | 1.89 | NA | NA | NA | NA | NA | NA |
| LEXOVE70 | alectinib | | PR | | 1.22 | 2.07 | NA | NA | NA | NA | NA | NA |
| LEXOVE71 | CT+ pembro | | PR | | 1.62 | 1.46 | NA | NA | NA | NA | NA | NA |
| LEXOVE72 | CT+ pembro | | PD | | 1.45 | NA | NA | NA | NA | NA | NA | NA |
| LEXOVE73 | CT | | PD | | 1.15 | 1.45 | NA | NA | NA | NA | NA | NA |
| LEXOVE74 | alectinib | | PD | | 0.64 | 1.9 | NA | NA | NA | NA | NA | NA |
| LEXOVE75 | osi | | PR | | 0.19 | 1.05 | NA | NA | NA | NA | NA | NA |
| LEXOVE77 | alectinib | | PD | | 0.36 | 1.01 | NA | NA | NA | NA | NA | NA |
| LEXOVE80 | CT | | PD | | 2.3 | 2.03 | NA | NA | NA | NA | NA | NA |

**Supplementary table S3.** Univariable and multivariable analysis for progression-free survival (PFS) in enrolled patients. Abbreviations: HR, hazard ratio; CI, confidence interval; Δ cfEV, cell-free extracellular vesicle protein levels from baseline to disease restaging.

| **PFS** | |  |  |  |  |
| --- | --- | --- | --- | --- | --- |
|  |  | **Univariate analysis** | | **Multivariate analysis** | |
| **Variable** | | **HR (95% CI)** | ***p*** | **HR (95% CI)** | ***p*** |
| **Gender** | Women | 1.0 (Reference) |  |  |  |
|  | Men | 2.01 (0.65-2.00) | 0.69 |  |  |
| **Age** | ≥ 65 | 1.0 (Reference) |  |  |  |
|  | < 65 | 1.54 (0,55 – 4,35) | 0.43 |  |  |
| **Ever smoker** | Yes | 1.00 (Reference) |  |  |  |
|  | No | 0.38 (0,09 – 1,71) | 0.21 |  |  |
| **Histology** | SCC | 1.00 (Reference) |  |  |  |
|  | Non-SCC | 0.73 (0,27 – 1,91) | 0.52 |  |  |
| **ECOG PS** | 0-1 | 1.00 (Reference) |  |  |  |
|  | **2** | **4.03 (1.45- 11.11)** | **0.007** | **4.03 (1.45- 11.11)** | **0.007** |
| **Treatment** | pembro | 1.00 (Reference) |  |  |  |
|  | others | 1.08 (0.39 – 3.04) | 0.88 |  |  |
| **Tissue PD-L1 (TPS)** | <1% | 0.53 (0.14 – 1.98) | 0.35 |  |  |
|  | 1-49% | 1.00 (Reference) |  |  |  |
|  | ≥50% | 0.96 (0.34 – 2.72) | 0.94 |  |  |
| Δ **EV cfEV** | ≥20% | 1.00 (Reference) |  |  |  |
|  | < 20% | 0.41 (0.15 – 1.11) | 0.08 |  |  |

**Supplementary table S4.** Association between clinical characteristics and circulating extracellular vesicle protein level (cfEV) dynamics in enrolled patients. Abbreviations: cfEV, cell-free EV protein levels; ECOG PS, Eastern Cooperative Oncology Group Performance Status; TPS, tumor proportion core; PFS, progression-free survival; OS, overall survival.

|  | | **Population** | | **p-value** | |
| --- | --- | --- | --- | --- | --- |
| **Characteristics** | | *N° cfEV<20% (%)* | *N° cfEV ≥20% (%)* |  |  |
| **Gender** | Men | 9 (69.2%) | 7 (63.6%) | 0.56 |  |
|  | Women | 4 (30.8%) | 4 (36.4%) |  |  |
| **Age (years)** | Mean ± Standard deviation | 69 +/-7.5 | 72 +/-9.6 | 0.41 |  |
|  | <65 | 2 (15.4%) | 3 (27.3%) | 0.41 |  |
|  | ≥65 | 11 (84.6%) | 8 (72.7%) |  |  |
| **Smoking habits** | Never smoker | 3 (23.1%) | 1 (9.1%) | 0.36 |  |
|  | Current or former smoker | 10 (76.9%) | 10 (90.9%) |  |  |
| **ECOG PS** | 0-1 | 9 (69.2%) | 5 (45.5%) | 0.22 |  |
|  | 2 | 4 (30.8%) | 6 (54.5%) |  |  |
| **Histological subtype** | Non-SCC | 8 (61.5%) | 10 (90.9%) | 0.12 |  |
|  | SCC | 5 (38.5%) | 1 (9.1%) |  |  |
| **Molecular diagnostics** | EGFR | 4 (30.8%) | 4 (36.4%) | 0.07 |  |
|  | ALK | 0 (0%) | 3 (27.3%) |  |  |
|  | ROS-1 | 0 (0%) | 1 (9.1%) |  |  |
|  | WT | 9 (69.2%) | 3 (27.3%) |  |  |
| **Tissue PD-L1 (TPS)** | Negative (<1%) | 2 (16.7%) | 3 (27.3%) | 0.66 |  |
|  | Low expression (1-49%) | 6 (50%) | 6 (54.5%) |  |  |
|  | High expression (≥50%) | 4 (33.3%) | 2 (18.2%) |  |  |
| **Site of disease** | Intra-thoracic | 5 (38.5%) | 4 (44.4%) | 0.56 |  |
|  | Extra-thoracic | 8 (61.5%) | 5 (55.6%) |  |  |
| **Treatment** | pembrolizumab | 4 (30.8%) | 1 (9.1%) | 0.07 |  |
|  | CT+/- pembrolizumab | 5 (38.5%) | 2 (18.2%) |  |  |
|  | ALK-TKI | 0 (0%) | 4 (36.4%) |  |  |
|  | osimertinib | 4 (30.8%) | 4 (36.4%) |  |  |
| **Radiologic response** | Complete response | 0 (0%) | 0 (0%) | 0.27 |  |
|  | Partial response | 9 (69.2%) | 5 (45.5%) |  |  |
|  | Stable disease | 2 (15.4%) | 1 (9.1%) |  |  |
|  | Progressive disease | 2 (15.4%) | 5 (45.5%) |  |  |
| **Progression** | Yes | 5 (38.5) | 2 (18.2%) | 0.26 |  |
|  | No | 8 (61.5%) | 9 (81.8%) |  |  |
| **PFS (months)** | Median (95% IC) | 25.23 (14.93 – 35.53) | 8.27 (3.60 – 12.94) | 0.07 |  |
| **Death** | Yes | 8 (61.5%) | 9 (81.8%) | 0.26 |  |
|  | No | 5 (38.5%) | 2 (18.2%) |  |  |
| **OS (months)** | Median (range) | 30.00 | 10.80 | 0.51 |  |
